# Estimating the magnitude of surveillance bias in COVID-19: Stefano Tancredi

**DOI:** 10.1093/eurpub/ckac130.096

**Published:** 2022-10-25

**Authors:** S Tancredi, S Cullati, A Chiolero

**Affiliations:** 1 Population Health Laboratory, University of Fribourg, Fribourg, Switzerland; 2 Department of Readaptation and Geriatrics, University of Geneva, Geneva, Switzerland; 3 Institute of Primary Health Care, University of Bern, Bern, Switzerland; 4 School of Population and Global Health, McGill University, Montreal, Canada

## Abstract

**Background:**

Most European countries implemented COVID-19 surveillance systems based notably on the number of diagnosed infections. Using this number as an indicator of epidemic severity is however problematic since it is influenced by testing modality. Indeed, differences in the frequency of infections are partly due to differences in detection rates rather than to changes in the risk of infection, leading to a “surveillance bias”. Our goal was to estimate the magnitude of this bias in one region of Switzerland, using population-based seroprevalence as the best marker of epidemic severity.

**Methods:**

We used data from serosurveys carried out on random samples of the adult population after the 1st (Jul-Oct 2020) and the 2nd wave of the pandemic (Nov 2020-Feb 2021), before the start of the vaccination campaign. To assess the scale of surveillance bias, we assessed the burden of COVID-19 between 2 waves comparing seroprevalence with the number of diagnosed cases (positive PCR or antigen tests).

**Results:**

Out of 867 participants (46% men), 8% (IC 95%:4%-12%) and 19% (IC:15%-23%) had anti-SARS-CoV-2 IgG after the 1st and 2nd wave respectively, that is, a 11% increase between waves. The cumulative number of SARS-CoV-2 diagnosed cases was 2'355 after the 1st wave and 23'321 after the 2nd, that is, an increase of 20'966 cases between waves. Based on the number of diagnosed cases, the epidemic severity of the 2nd wave was 8-9 times higher compared with the 1st wave (20'966 vs 2'355 cases). Based on seroprevalence estimates, epidemic severity of the 2nd wave was less than 1.5 times higher compared to the 1st wave (11% vs 8%).

**Conclusions:**

Due to changes in testing modalities, the number of cases is problematic to assess the burden of COVID-19 in different phases of the pandemic. Accounting for surveillance bias is necessary for accurate public health surveillance.

**Key messages:**

- Accounting for surveillance bias and critically interpreting surveillance data is essential for an accurate public health monitoring activity.
- The number of diagnosed cases cannot be used alone to assess the burden of COVID-19.

